# Neonatal eyelid penetration from insertion of a fetal scalp electrode: a case report

**DOI:** 10.1186/s12884-022-05146-4

**Published:** 2022-11-26

**Authors:** Brian T. Cheng, Kelly D. Laurenti, Sudhi P. Kurup

**Affiliations:** 1 Department of Ophthalmology, Northwestern University Feinberg School of Medicine, Chicago, IL USA; 2 Division of Ophthalmology, Ann & Robert H. Lurie Children’s Hospital of Chicago, 225 E. Chicago Ave., Box 70, Chicago, IL 60611 USA

**Keywords:** Pediatric ophthalmology, Obstetric complication, Fetal scalp electrode, ophthalmic injury, Case report

## Abstract

**Background:**

A fetal scalp electrode (FSE), first described by Edward Hon in 1967, is an intrapartum monitoring device embedded directly into the fetal scalp for an accurate measure of fetal heart rate. Though use of an FSE is generally safe, complications can occur from misplacement, including ophthalmic injury.

**Case presentation:**

Patient was a 28-year-old G6P5006 who presented for induction of labor at 39 weeks due to asymptomatic bilateral pulmonary embolism. Concerning findings on external fetal monitoring led to placement of a fetal scalp electrode for close monitoring. Upon delivery, the neonate was noted to have the FSE embedded in the left upper eyelid. Ophthalmology was consulted and could not rule out ocular injury on external examination at the bedside. Examination under anesthesia in the operating room demonstrated no penetration of the ocular globe, and the eyelid laceration was sutured. The laceration was well-healing at one-week follow-up with no further complications.

**Conclusion:**

Facial or brow presentation during delivery is rare but may increase the risk for misplacement of an FSE. Ultrasound verification of vertex position is warranted immediately prior to placing an FSE for patients at higher risk of facial or brow presentation. Periorbital edema of neonates may protect against damage to deeper structures. However, Ophthalmology should be consulted to rule out ocular injury if the FSE is placed in the periocular region.

## Background

A fetal scalp electrode (FSE) is a sensor that is placed directly on the fetal scalp to obtain reliable intrapartum assessment of the fetal heart rate. The FSE contains a spiral wire tip that must be screwed into the fetal scalp. Previous studies have reported on complications stemming from its placement, including abscess, infection, and cephalohematoma [1, 2]. Here, we present the unique clinical course of a neonate in whom an FSE was placed in the left upper eyelid during delivery.

## Case presentation

The mother was a 28-year-old, G6P5006 female who presented at 39 weeks for induction of labor due to active, asymptomatic bilateral pulmonary embolism with a history of recurrent pulmonary embolism and deep vein thrombosis. She previously had two spontaneous vaginal deliveries, two Caesarean sections, and two successful vaginal births after Caesarean section. Vertex presentation was confirmed by ultrasound, and labor was induced. At 3–cm cervical dilation, external fetal monitoring was concerning. Therefore, oxytocin was stopped, and a single helix FSE (Covidien Kendall™; Dublin, Ireland) was placed for better continuous monitoring of the fetus. Review of the record did not comment on the verification of fetal position immediately prior to FSE placement. A baby boy (birth weight = 3.255 kg) was delivered vaginally in left occiput anterior position with Apgar scores of 8 and 9 at 1 and 5 minutes, respectively.

**Fig. 1:**
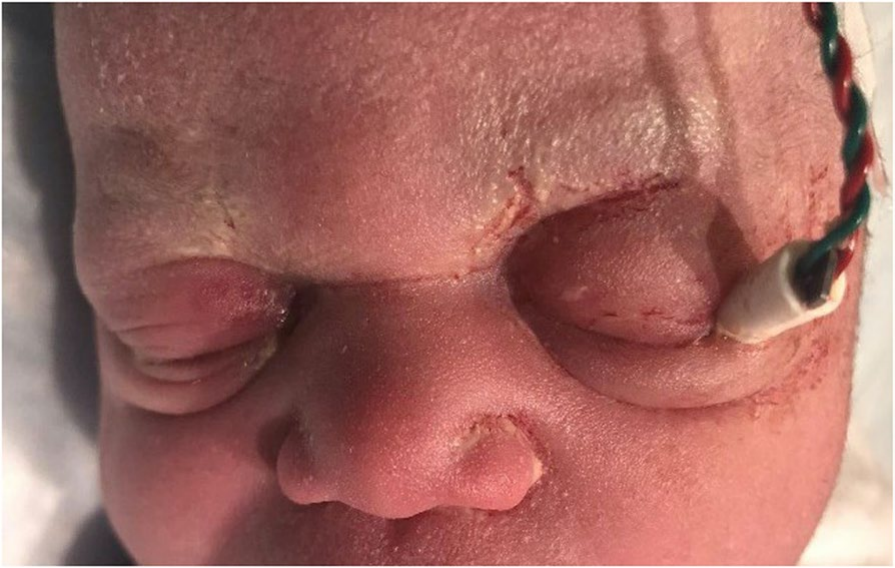
External photo of neonate three hours postpartum at bedside showing periorbital edema (left > right) and the FSE embedded in the left upper eyelid at the lateral canthus

Upon delivery of the neonate, the FSE was noted to be embedded in the left upper eyelid. Ophthalmology was consulted. On external examination, bilateral upper eyelids demonstrated marked edema consistent with postnatal swelling but no ecchymosis. The FSE was embedded in the left upper eyelid near the lateral canthus with adjacent superficial laceration but without significant discharge or bleeding (Fig. 1). Given the eyelid swelling and position of the FSE, bedside exam could not rule out ocular injury, so the neonate was urgently taken to the operating room.

**Fig. 2:**
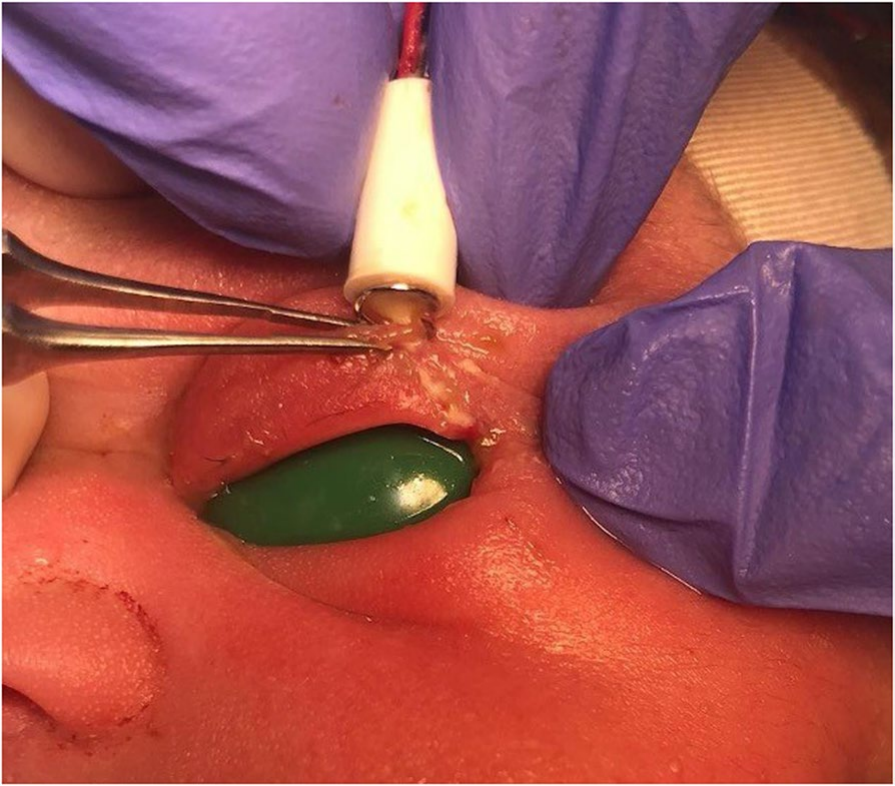
External photo of neonate in the operating room with the FSE embedded in the retracted left upper eyelid

In the operating room, gentle manipulation allowed for view of an intact ocular surface and no evidence of full-thickness penetrating injury by the FSE (Fig. 2). A scleral shell was placed over the ocular surface, and the FSE was removed from the eyelid by unscrewing in a counter-clockwise fashion. The remaining examination was unremarkable. The laceration was repaired with 7-0 Vicryl and treated with ophthalmic erythromycin ointment; wound healing was uncomplicated at one-week follow-up.

Written, informed consent was obtained at the follow-up appointment for the publication of this case and accompanying images.

## Discussion and conclusion

Fetal scalp electrodes (FSE) are commonly used to obtain more accurate fetal monitoring as an alternative to external Doppler. An estimated 22% of deliveries involve placement of an FSE [1]. The FSE is especially useful for continuous monitoring in cases of non-reassuring fetal heart tones or large body habitus. Contraindications for FSE include fetal facial or brow presentation, intact fetal membranes, placenta previa, infection with human immunodeficiency virus (HIV), active herpetic lesions, and other infectious risks to the fetus [3]. The distal electrode with a spiral wire tip must be screwed into and penetrate the scalp. The most frequently reported complications of FSE use include cellulitis, abscess, sepsis, and cephalohematoma and meningitis in the fetus, and endometritis, chorioamnionitis, and vaginal or cervical trauma in the mother [1, 2]. Another possible complication is placement of the FSE in the periorbital region, which may result in intraocular injury [4]. We describe a patient delivered in the cephalic left occipital anterior position with an FSE embedded in the left upper eyelid during delivery who fortunately recovered without complication. Periorbital edema in the neonate likely protected the infant from complete eyelid penetration, globe injury, or deeper ocular damage. However, Ophthalmology should be consulted if the FSE is embedded in the periocular region or if there is concern for periocular injury (e.g. laceration or ocular swelling/redness) caused by FSE misplacement, for thorough ophthalmic examination to rule out ocular injury.

Facial or brow presentation of the fetus also increases the likelihood of ocular complications secondary to FSE placement. Risk factors for facial and brow presentations include multiparity and previous Caesarean section [5]. Lower abdominal muscle tone in multiparous patients may lead to pendulum swinging of the fetus' abdomen forward, extending the neck and increasing the likelihood of facial or brow presentation [6]. Delayed engagement of the fetal head and maternal pelvis in multiparous women may also contribute to higher rates of facial or brow presentation. It has been suggested that previous Caesarean section may cause lower uterine segment contractile dysfunction that limits head flexion during delivery [5]. For patients with multiple risk factors for facial or brow presentation, fetal monitoring is ideally performed with an external probe. However, previous studies suggest there is a high prevalence of severe variable decelerations and late decelerations associated with facial presentation that may necessitate an internal device [7, 8]. As such, for patients at greater risk for facial or brow presentation and requiring internal monitoring, clinicians should verify vertex position immediately prior to FSE placement to reduce the likelihood of FSE misplacement, as most cases of facial and brow presentation are not diagnosed until the second stage of labor [9]. In the presented patient case, vertex position had been visualized on ultrasound prior to induction, but review of the medical record did not document that an ultrasound was repeated before FSE placement. If internal fetal monitoring is required for cases of facial or brow presentation, extreme caution should be taken to apply the FSE over forehead, mandible, or other bony structure to avoid injury.

Two previous cases of ocular adnexal injury by an FSE have been reported in ophthalmology literature. The first involved a neonate with an FSE that was inadvertently placed on the left upper eyelid during labor and avulsed by its own weight during caesarean section; further ocular examination identified superficial eyelid lacerations but no globe injury [10]. The second report detailed a case in which the FSE was placed on the right eye, and further examination found that the FSE had penetrated the inferior sclera and torn the peripheral retina [4]. The patient eventually developed lens dislocation and required complete removal of the lens and capsule and anterior vitrectomy three years after initial injury; at eight years of age, visual acuity was 20/200 with a contact lens in the injured eye and 20/20 without correction in the contralateral eye [4].

In conclusion, facial presentation occurs in 1 in 600 births, and brow presentation has a prevalence of 1 in 500-4000 births [11]. Though uncommon, facial or brow malpresentation can increase the risk for misplacement of the FSE. This case report highlights the importance of confirming the fetal position prior to placement of the FSE. If there is suspicion for facial or brow presentation of the fetus, clinicians should avoid using an FSE, if possible, or take extreme care in the placement. Ophthalmology consult is warranted in the event of periorbital injury caused by FSE placement to exclude deeper ocular injury.

## Data Availability

Data sharing not applicable to this article as no datasets were generated or analyzed during the current study.

## Abbreviations

FSE: Fetal Scalp Electrode
HIV: Human Immunodeficiency Virus
